# Editorial: Genome editing and biotechnological advances for crop improvement and future agriculture

**DOI:** 10.3389/fpls.2023.1132821

**Published:** 2023-01-31

**Authors:** Mohd Fadhli Hamdan, Goetz Hensel, Anshu Alok, Boon Chin Tan

**Affiliations:** ^1^ Centre for Research in Biotechnology for Agriculture, Universiti Malaya, Kuala Lumpur, Malaysia; ^2^ Centre for Plant Genome Engineering, Institute of Plant Biochemistry, Heinrich-Heine-University, Dusseldorf, Germany; ^3^ Centre of Region Haná for Biotechnological and Agricultural Research, Czech Advanced Technology and Research Institute, Palacký University Olomouc, Olomouc, Czechia; ^4^ Department of Plant Pathology, University of Minnesota, Saint Paul, MN, United States

**Keywords:** genetic engineering, genome editing, biotechnology, crop improvement, CRISPR, omics

Climate change-induced environmental stresses, pests, and diseases significantly affect crop growth and productivity, posing severe threats to food security. Given the increasing population and environmental pressures, it is essential to develop crops resilient to environmental stresses and capable of sustained yield and nutrition (Ghag et al., 2022). While conventional crop breeding can help develop new varieties with improved traits, it is a slow, labor-intensive, and costly process (Lau et al., 2022). Modern biotechnology and genome editing technologies could overcome these limitations and accelerate plant breeding beyond what was previously imaginable. Integrating high-throughput omics technologies, reinforced by next-generation sequencing, and the advanced genome editing tools allows for precise manipulation of crop genomes, enabling the development of varieties with desired traits and improved adaptability to changing climates. Additionally, the safe deployment of these technologies in crop improvement may aid in achieving the United Nations’ goal of ending global hunger. In this scenario, this Research Topic compiles five articles (one review and four original research articles) that explore the latest technological advances and applications of genome editing and/or omic technologies in crop improvement.

Among the recent genome editing technologies, the clustered regularly interspaced palindromic repeat (CRISPR)/CRISPR-associated (Cas) protein (CRISPR/Cas) is the potent and widely adopted tool for genome editing in plants. The CRISPR/Cas system consists of the Cas endonuclease and a synthetic single-guide RNA (sgRNA) that directs the Cas protein to a targeted genomic DNA (gDNA) sequence, which is then recognized and cleaved by CRISPR/Cas9 (Karlson et al., 2021; Hamdan et al., 2022). There are several Cas endonucleases, such as Cas9, Cas12a, Cas12b, Cas12j (Casϕ), and Cas12f (CasMINI) (Alok et al., 2021). The cleavage of DNA can often result in insertions or deletions (InDels), which can lead to frameshift mutations and gene knockouts. CRISPR/Cas9 has been widely used for genome editing in various plant species, including model plants, cereal crops, oil crops, fruits, vegetables, and horticulture plants (Okamoto et al., 2022; Yao et al., 2022). However, genome editing in vegetables is limited, and till now, radish genome editing has been unexplored. In this special issue, Muto and Matsumoto used a CRISPR/Cas9 system with a sgRNA to modify the *GLABRA1* (*GL1*) orthologs, *RsGL1a* and *RsGL1b*, which are known to be involved in leaf trichome formation in radish. The authors found that at least 62% editing efficiency was recorded in T0 plants, and most of the mutant alleles were stably inherited in the T1 generation. This study highlighted that genome-edited radish without T-DNA (null-segregant) could be obtained as novel breeding material. This could have significant implications for crop improvement and agriculture, as it may provide a more efficient and cost-effective way to develop new radish varieties with improved traits. Furthermore, the developed technology is expected to apply to other vegetables and traits, including resistance to pests and diseases, improved nutritional value, and tolerance to environmental stresses, which is crucial in addressing food security and sustainable agriculture in the face of climate change.

In another study, Cao et al. employed a multiplex CRISPR/Cas9 approach in soybean to target *RS2* and *RS3* genes responsible for raffinose synthesis. They showed that the two-component transcriptional unit (TCTU) and single transcriptional unit (STU) systems with tRNA as the cleavage site showed better editing efficiency than other systems. Furthermore, using the TCTU-tRNA system, Cao et al. successfully induce mutations at *RS2* and *RS3*, resulting in significant reductions in raffinose family oligosaccharides and high sucrose levels in soybean. Their findings suggest that the multiplex CRISPR/Cas9 approach could be a promising way to improve the quality of soybeans for human and monogastric animal consumption.

The application of CRISPR/Cas technology has shown promising potential in maize crops. Wang et al. summarize the current applications and potential of CRISPR/Cas technology in maize, focusing on gene function and generating new germplasm for increased yield, specialty corns, plant architecture, stress response, haploid induction, and male sterility. The review also highlighted significant obstacles that limit the use of CRISPR technology in maize, such as low gene editing frequency, low genetic transformation efficiency, and regulatory issues. This indicates that technological advances are needed and that existing regulatory policies should be revisited to fully realize the potential of CRISPR/Cas technology in maize crop improvement.

Plant pathogens can significantly reduce crop yield and quality, which poses a significant threat to global food security (Ghag et al., 2022). To address this issue, it is necessary to enhance the genetic resistance of plants to diseases through sustainable agricultural production methods. The work by Cheng et al. demonstrates the feasibility of using CRISPR/Cas9 genome editing to accelerate breeding for disease resistance in barley. The authors modified *Protein Disulfide Isomerase Like 5-1* (*PDIL5-1*), which is known to play a role in the plant’s response to viral infections in barley. The study found that the mutated plants were more resistant to yellow mosaic virus disease than wild-type plants, with no adverse effects on growth or yield. This enhanced resistance may lead to more sustainable agricultural practices by mitigating the negative impacts of plant pathogens on global food production.

Aphids are common insect pests that can cause significant crop yield losses, ranging from 25 to 30% under severe infestation (Jasrotia et al., 2021). To investigate the aphid-host interactions, Jasrotia et al. analyzed the transcriptome changes in two wheat genotypes: a highly susceptible wheat *Triticum durum* genotype (A-9-30-1) and a tolerant *Triticum aestivum* genotype (HD2967) before and after infestation by corn leaf aphids. The study found that the infestation caused significant changes in the transcriptome of the wheat plants, with genes involved in defense being significantly more expressed in HD2967 compared to A-9-30-1. These findings provide insights into the defense mechanisms of wheat plants against aphids and could potentially lead to the development of resistant varieties. This is especially important for future agriculture, as reducing yield losses caused by aphid infestations is crucial for ensuring food security and sustainability.

Overall, the five articles in this Research Topic highlight the potential of genome editing and biotechnologies advances to develop crops with improved traits, which will be essential for addressing future food security challenges.

## Author contributions

All authors listed have made a substantial, direct, and intellectual contribution to the work and approved it for publication.
